# Dimethyl fumarate as a versatile therapeutic agent: molecular mechanisms and potential clinical applications

**DOI:** 10.1007/s11033-026-11478-7

**Published:** 2026-02-02

**Authors:** Eleni Zingkou, Alessandro Medoro, Sergio Davinelli, Luciano Saso, Georgia Sotiropoulou, Georgios Pampalakis

**Affiliations:** 1 Department of Pharmacy, University of Patras, 26504 Rion-Patras, Greece; 2 Department of Medicine and Health Sciences “V. Tiberio”, University of Molise, Campobasso, Italy; 3 Department of Physiology and Pharmacology, Vittorio Erspamer”, Sapienza University, Rome, Italy; 4 Laboratory of Pharmacology, School of Pharmacy, Aristotle University of Thessaloniki, 54124 Thessaloniki, Greece

**Keywords:** Dimethyl fumarate (DMF), Drug repurposing, Inflammation, Neuroinflammation, Skin diseases, Cardiovascular, Pulmonary

## Abstract

Dimethyl fumarate (DMF) is an electrophilic compound used clinically for multiple sclerosis and psoriasis. We elaborate here that the pharmacological effects of DMF extend beyond the well-known activation of the NRF2 antioxidative pathway. Indeed, DMF directly modifies reactive cysteine residues on multiple proteins in immune and neural cells, leading to diverse anti-inflammatory, immunomodulatory, and neuroprotective actions. Recent studies revealed that DMF may affect proteins involved in inflammasome activation, glycolysis, and cell signaling pathways, including JAK-STAT and NF-kB. These effects may expand the potential clinical applications of DMF in diverse pathologies, including neurodegenerative, cardiovascular, and pulmonary diseases. We summarize current findings on chemical reactivity, target proteins, and emerging clinical applications of DMF, highlighting new opportunities for DMF repurposing.

## Introduction

Dimethyl fumarate (DMF) is a simple α,β-unsaturated organic molecule with a highly electrophilic character that readily reacts with nucleophiles through Michael addition. The use of DMF in therapeutics was introduced by the German chemist Walter Schweckendiek in 1959 with the purpose to treat his own psoriasis [1]. DMF was initially approved by the US Food and Drug Administration (FDA) and the European Medicines Agency (EMA) under the trade name Tecfidera® for the treatment of Relapsing–Remitting Multiple Sclerosis (RRMS). DMF in a mixture with other fumarates [calcium, magnesium, and zinc salts of monomethyl fumarate (MMF)] was approved in Germany in 1994 under the trade name Fumaderm® for treatment of severe psoriasis and extended in 2008 for moderate psoriasis. In EU, DMF was approved in 2017, under the trade name Skilarence®, for treatment of moderate to severe plaque psoriasis [2].

DMF is thought to act through activation of the nuclear factor erythroid 2-related factor 2 (NRF2) pathway. Specifically, DMF covalently modifies the cysteine residue of the Kelch-like ECH-associated protein 1 (KEAP1) via succination. Succination of KEAP1 results in dissociation from its complex with NRF2, allowing for activation of the NRF2 [3]. On July 24th, 2025, the information document for Tecfidera® available by EMA indicated that the pharmacological action of DMF in RRMS, is through the activation of the NRF2 (https://www.ema.europa.eu/en/documents/product-information/tecfidera-epar-product-information_en.pdf). Nevertheless, it is widely recognized that DMF exerts multiple roles that include antitumor, antioxidant, anti-inflammatory, neuroprotective, antiangiogenic, and immunomodulatory actions, which cannot be solely attributed to NRF2 activation.

Importantly, the involvement of other mechanism(s) accounting for the pharmacological efficacy of DMF was based on the observation that DMF showed equal clinical benefit in acute experimental autoimmune encephalomyelitis (EAE) in both *Nrf2*^+*/*+^ and *Nrf2*^*−/−*^ mice [4]. Indeed, recent data have unraveled new pathways that are modulated by DMF and may even be more important in determining its pharmacological efficacy over the DMF-induced activation of the NRF2 pathway. Here, we present data support the implication of new alternative molecular mechanisms accounting for the enhanced pharmacological efficacy of DMF. Further, we suggest the exploitation of DMF for the treatment of a large variety of diseases that extend from its classical use in skin and neuroinflammatory diseases to cardiovascular and pulmonary diseases and even cancer.

## Chemistry of DMF

**Fig. 1:**
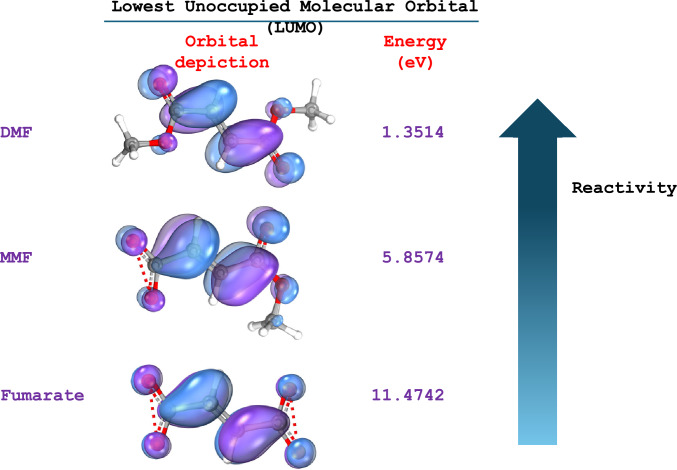
The structure of fumarates was designed with Avogadro and energy minimization was carried out. The data were exported in Orca format, and the HOMO and LUMO energies were calculated with Orca. Visualization of molecular orbitals was conducted with IboView.

Within the body, DMF is rapidly converted to MMF with a half-life of 12 min, while the MMF has a half-life of 36 h [1]. For this reason, it was considered that DMF was a prodrug. However, recent data suggest that DMF is not a prodrug, rather it is an active drug that may even be more efficacious than MMF. Its greater efficacy relative to MMF (or even fumarate), could be conferred by its enhanced chemical reactivity. Specifically, Fig. 1 shows the calculated Lower Unocuppied Molecular Orbital (LUMO) energies, that is only 1.35 eV for DMF, indicating a highly reactive compound, while MMF and fumarate show LUMO energies of 5.86 and 11.47 eV, respectively. Moreover, the absence of charge allows DMF to freely enter the cells to reach with intracellular targets.

**Fig. 2:**
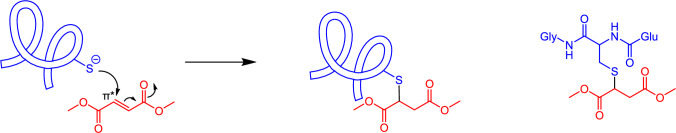
Chemical reaction of DMF with thiols (succination) (left) and structure of a GSH-DMF adduct (right)

The modification of free thiols in proteins by DMF is referred to as succination of proteins. In addition, DMF readily reacts with glutathione (GSH) and depletes it, thus perturbing redox homeostasis in the cell. This may provide an additional mechanism that explains the efficacy of DMF [5] (Fig. 2). To this end, it should be mentioned that fumarate is also produced in the Krebs cycle and is responsible for the endogenous succination, to be discussed below. DMF analogs were also developed like the diroximel fumarate (Vumerity®) that displays improved gastrointestinal tolerability, which was approved by the FDA for treatment of RRMS [6]. Furthermore, the tepilamide fumarate is now in Phase IIb clinical trials for treatment of moderate-to-severe plaque psoriasis [7].

## Methods for identification of the DMF targetome

As mentioned, the therapeutic efficacy of DMF was mainly attributed to KEAP1 succination, which resulted in stabilization of NRF2 leading to activation of the antioxidant response element (ARE). Nevertheless, multiple additional mechanisms of action of DMF occur independent of NRF2 activation, indicated by the recorded efficacy of DMF in diseases, like RRMS and psoriasis, which points to a strong immunomodulatory effect. Thus, the identification of the DMF targetome becomes of high interest as it may provide clues to alternative biological pathways of DMF action and could identify yet unknown DMF targets, leading to the development of more specific therapeutic approaches with enhanced therapeutic efficacy. The methods for the characterization of the DMF targetome are based on mass spectrometry and are outlined below.

### Direct detection of succinated sites

Cells are treated with DMF (or MMF or fumarate) or the solvent, and the proteins are collected, reduced with dithiothreitol and treated with 4-vinylpyridine to convert the non-DMF labeled cysteines (Cys) to pyridylethylated Cys. Then, the proteomes are trypsinized and the resulting proteolytic peptides are analyzed with Liquid Chromatography/Tandem Mass Spectrometry (LC–MS/MS) for identification of modified Cys residues. The reaction Cys residues with DMF yields a 2-dimethyl succinyl-derivative of Cys, the reaction with MMF, a 2-monomethyl succinyl-derivative and the reaction with fumarate, a 2-succinyl-derivative. The identified peptide fragments carrying modified Cys residues allow the positional mapping of the electrophile susceptible Cys in the parent proteins [8].

### Use of activity-based proteomics

**Fig. 3:**
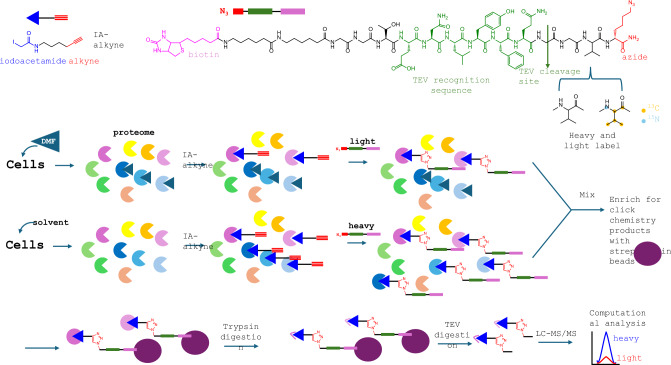
Schematic representation of the isoTOP-ABPP methodology used to map succinated Cys residues

The method is known as isotopic Tandem Orthogonal Proteolysis-Activity-Based Protein Profiling (IsoTOP-ABPP) and is based on the competition between DMF and a general electrophilic probe, the iodoacetamide-alkyne (IA-alkyne). Cells are incubated with DMF or the appropriate solvent. The proteins are extracted and treated with IA-alkyne probe which competes with the DMF sites. Then, a copper catalyzed azide-alkyne 1,3 dipolar cycloaddition (CuACC) is carried out with an azide compound to facilitate labeling. Briefly, the solvent (control) reaction is allowed to react with an azide compound bearing a heavy isotope labeled tag separated with a TEV (tobacco etch virus) protease-cleavable site from a biotin moiety, while DMF with the low isotope respective azide compound [9] (Fig. 3). The DMF and solvent treated samples are mixed in equal amounts, enriched with streptavidin beads, and subjected to sequential digestion with trypsin, to generate the fragments for MS analysis, and to TEV to release the labeled Cys-containing peptides from the streptavidin beads.

Important features of the isoTOP-ABPP are: (a) its ability to use non-modified electrophilic compounds (like DMF), and (b) isotopic labeling is carried out later during sample processing, thus, it provides accurate quantitative analysis without risk for potential alterations due to metabolic labeling.

Previously it was shown that T cells from both wild-type and *Nrf2*^*−/−*^ mice were sensitive to inhibition by DMF, indicating that DMF exploited a NRF2-independent pathway. isoTOP-ABPP identified protein kinase C θ (PKCθ) as a DMF target. Specifically, the residues Cys14 and Cys17 in PKCθ, located within the CXXC motif that is critical for its interaction with CD28 to induce T cells activation were mapped [9]. Succination of these Cys residues prevents interactions of PKCθ with the costimulatory receptor CD28, thereby preventing T cell activation. MMF cannot modify these residues, in accordance, it does not act as an inhibitor of T cell activation. Mutant PKCθ (Cys14Ser and Cys17Ser) could not drive the activation of T cells, which confirms the crucial role of these Cys residues in T cell activation.

The simplified and ultrafast peptide enrichment and release (superTOP-ABPP) method represents a recent improvement over isoTOP-ABPP, which utilizes agarose beads functionalized with azide groups and acid-cleavable linkers [10]. Thus, it takes one step from click chemistry reaction and peptide enrichment for mass spectrometry. To this end, it should be mentioned that the analytical methods that rely on “clickable” tags based on biorthogonal chemistry are advantageous, since they avoid reactions with activity-based probes carrying bulky biotin or fluorescent moieties that may severely affect the binding with the target protein.

### Use of DMF-based activity-based probes

This method highly resembles the previously described, but it uses an alkyne modified DMF, the DMF-yne [11]. Nevertheless, using the antioxidant response element (ARE) luciferase assay, it was shown that DMF-yne and DMF have comparable potency for NRF2 activation, indicating that the addition of alkyne does not greatly affect the properties of DMF. Furthermore, both compounds lack charge and are cell permeable. The cells (or lysates) are incubated with the DMF-yne or the solvent. Then, the modified proteins are biotin-labeled with click chemistry and enriched with streptavidin beads. Subsequently, the labeled proteins are trypsinized and labeled with Tandem Mass Tag (TMT) reagents, mixed, and analyzed by LC–MS/MS. The TMT label is also known as isobaric label, since it yields equal m/z during the MS^1^ analysis, but during the MS/MS or MS^2^ the differences become apparent, since the TMTs carry linkers that are cleavage with the high-energy collision-induced dissociation, to allow quantification of proteins in different samples [12]. It is interesting to note that DMF-yne labeled many proteins in SH-SY5Y cells as revealed by Western blot, while MMF-yne showed minimal staining, further supporting the notion that DMF is the active pharmacological compound [11].

## Protein and pathways regulated by DMF

Using various methodologies for mapping the DMF targetome, several targets containing active thiols have been found, as shown in Table 1. The reactivity of one Cys residue over another Cys residue, within the same protein, depends on the local environment and the pKa of the specific Cys. For the succination reaction to take place, the pKa of the thiol must be low, so that the thiol will be present in its deprotonated, *i.e.*, the nucleophilic form.

**Table 1:** Proteins subjected to succination by DMF or MMF

Protein	DMF/MMF	Residue	System	Notes	References
KEAP1	DMF/MMF	Cys^151^ (major) Cys^257^, Cys ^273^ (minor) (human)	In vitro in HEK293	DMF is more potent than MMF MMF succinates only C^151^	[13]
GAPDH	DMF/MMF	Cys^152^ (active site) Cys^156,247^ (human) Cys^150^ (active site) (mouse)	In vitro purified protein In vivo in mice and in PBMCs from human RRMS patients treated with DMF	DMF is more potent than MMF In PBMCs only Cys152 was succinated by DMF	[24]
GSDMD	DMF/MMF	Cys^191^ (human) Cys^192^ (mouse) Other minor Cys succination sites were determined	In vitro purified protein and BMDM treated with DMF	MMF showed no activity in blocking secretion of cytokines from primed BMDM, most likely due to impermeability Diroximel and tepilamide fumarate also blocked pyroptosis and formation of GSDMD-N	[15]
GSDME	DMF/MMF	Cys^45^ (mouse) Other minor sites were determined	In vitro in BMDM mouse		[15]
IKK2	DMF	Cys^179^ (kinase domain) C^464^ (leucine zipper domain) C^347^ (ubiquitin-binding domain of NEMO) (human)	In vitro in DMF treated ABC-diffused large B-cell lymphoma	C^179^ the important for the activity of the IKK2	[19]
JAK1	DMF	Cys^257^, Cys^731^ (human)	In vitro in DMF treated ABC-Diffused large B-cell lymphoma		[19]
HCAR2	DMF/MMF	AGONISTS	In vivo in mouse models		[29]
PRKCQ (PKCθ)	DMF/MMF	Cys^14^, Cys^17^ (human, mouse)	In vitro DMF treated activated T cells	Only DMF reacts	[9]
PRKDC	DMF	Cys^4045^ (human)	In vitro DMF treated activated T cells		[9]
COFILIN-1	DMF/MMF	Cys^139^ (mouse, rat)	In vitro mouse and rat neuronal cells In vitro rat astrocytes		[17]
Tubulin α and β dimer	DMF	Cys^347^ (mouse, rat)	In vitro mouse and rat neuronal cells In vitro rat astrocytes		[17]
Tubulin α and β dimer	DMF/fumarate	Cys^295^, Cys^315^, Cys^316^, Cys^347^, Cys^376^ in α-tubulin, and Cys^12^, Cys^127^, Cys^129^, Cys^239^, Cys^303^, Cys^354^ in β-tubulin (porcine, DMF) Cys^347^, Cys^376^ in α-tubulin and Cys^12^, Cys ^303^ in β-tubulin (porcine, DMF)	In vitro purified porcine tubulin In vitro mouse fibroblasts, adipocytes, myocytes	DMF is much more reactive in vitro than fumarate	[18]
NLRP3	DMF	Cys^673^ (human)	In vitro in THP1-cells		[21]
NEK7	DMF	Cys^298^ (mouse)	In vitro in mouse BMDM	An indirect methodology was used to map the modified residue	[22]
IRAK4	DMF DMF/MMF	Cys^13^ (human)	In vitro in Cal-1 cells In vitro in mixed Ramos and Jurkat cells	MMF reacted with IRAK4 at 5 times higher concentration relative to DMF	[11, 30]
p65	DMF	Cys^38^ (human)	In vitro in MDA-MB-231 cells	An indirect methodology was used to map the modified residue	[81]

PBMC: peripheral blood mononuclear cells

BMDM: bone marrow–derived macrophages

### DMF activates NRF2

This is the most classic reaction described for DMF. KEAP1 is a ubiquitin E3 ligase that is responsible for degradation of NRF2. DMF was determined to react with Cys151, Cys273, and Cys288 of KEAP1. In contrast, MMF reacts only with Cys151 [13]. Using transgenic mice expressing mutants of KEAP1, it was found that Cys273 and Cys288 are required for NRF2 activation in vivo. The Cys151 was not critical for NRF2 activation, since Tg-*Keap1*^*Cys151Ser*^ mice could rescue the lethal phenotype of *Keap1*^*−/−*^ mouse, *i.e.*, they could reverse the constitutive activation of NRF2 [14]. The fact that MMF succinates the Cys151 that is not important for the inhibitory action of KEAP1, supports the idea that DMF is the “true” drug and not the prodrug of MMF.

### DMF succinates gasdermin D (GSDMD)

GSDMD is a precursor of a pore forming protein, and its inactivation blocks pyroptosis. Succination of GSDMD prevents its interaction with caspase-1 that liberates the *N*-terminal domain of GSDMD (GSDMD-N), which is the active form amenable to oligomerization and formation of pores in the plasma membrane. Also, DMF inhibits the oligomerization of GSDMD-N. Inactivation of GSDMD by DMF has been suggested as a mechanism accounting for the therapeutic efficacy of DMF in MS [15] which is corroborated by findings that *Gsdmd*-deficient mice are protected from experimental autoimmune encephalitis (EAE) [16]. The importance of GSDMD in driving MS is also indicated by the fact that patients with MS have elevated GSDMD-N levels in their peripheral blood mononuclear cells (PBMCs) [15].

### DMF succinates cytoskeletal proteins and affects their function

Cofilin-1 is a cytoplasmic protein that depolymerizes actin filaments (F-actin), thus, it controls the actin cytoskeleton. Succination of cofilin-1 at Cys139 by DMF, renders it incapable of depolymerizing F-actin in neuronal cells [17].

The αβ-tubulin dimer contains a total of twenty Cys residues, twelve in the α- and eight in the β-tubulin monomer. DMF reacts with eleven Cys residues (Table 1) and reduces tubulin polymerization. Fumarate also reacts with αβ-tubulin but at 100 times higher concentration relative to DMF and with reduced efficiency [18]. Nevertheless, succination of tubulin does not affect the axonal trafficking of lysosomes [17]. The reaction with fumarate suggests that increased levels of endogenous fumarate, as found in certain disease conditions like the aggressive form of kidney cancer known as hereditary leiomyomatosis and renal cell carcinoma (HLRCC), may affect microtubule dynamics.

### DMF directly interferes with the Janus kinase/Signal transducers and activators of transcription (JAK/STAT) pathway

Diffuse large B-cell lymphoma (DLBCL) is the most common malignant lymphoma in adults. DMF shows antitumor activity in both subtypes of DLBCL, namely, the germinal center B cell (GCB)-like and the activated B cells (ABC) DLBCL. In GCB-DLBCL, DMF induces ferroptosis through depletion of GSH and upregulation of arachidonate 5-lipoxygenase (5-ALOX). In ABC-DLBCL, DMF succinates the Inhibitor Nuclear Factor kappa-B kinase 2 (IKK2) and JAK1 to inhibit both NF-kB and JAK/STAT survival signaling [19]. The authors found that the action of DMF was independent on NRF2 signaling. In IKK2, succination of the following residues was found: Cys179 (located in the kinase domain), Cys464 (located in the leucine zipper domain), and Cys347 (located in the NEMO domain) of which modification of Cys179 was shown to be important for inhibition of IKK2 signaling by DMF. Further, DMF inhibited STAT3 and STAT1 phosphorylation in ABC-DLBCL, which led to the hypothesis that DMF could alter the activity of JAKs. Indeed, DMF reacted with JAK1, JAK2, and tyrosine kinase 2 (TYK2), but the authors only analyzed the effect of DMF on JAK1. It was shown that DMF succinated Cys257 and Cys731, of which the Cys257 is important for interaction of JAK1 with cytokine receptors. In accordance, it was demonstrated that DMF inhibited the autophosphorylation of JAK1 and TYK2 [19].

MMF was inert and could not inhibit IKK2 activity or the phosphorylation of STAT3 [19], also suggesting that DMF is the “true” drug. Modification of JAKs by DMF may provide an explanation for the recent finding that DMF inhibited the Jak2/Stat3 pathway to ameliorate pyroptosis in a mouse model of an acute renal injury [20].

### DMF prevents inflammasome activation

DMF inhibited the early activation of NLRP3 and the formation of the NLRP3-inflammasome that plays important roles in various chronic inflammatory conditions. Specifically, DMF succinates NLRP3 Cys673, thus, preventing its interaction with NEK7. This interaction is required to facilitate the interaction of NLRP3 with ASC to promote the assembly and activation of the inflammasome. The importance of this pathway was demonstrated in vivo, where DMF was shown to relieve the symptoms of dextran sodium sulfate (DSS)-ulcerative colitis model through inhibition of the NLRP3 inflammasome. The effect of DMF was not noticed in *Nlrp3*^*−/−*^ mice [21]. Very recently, it was also shown that DMF succinates the Cys298 residue of NEK7 that also inhibits its interaction with NLRP3 [22]. To this end, it should be mentioned that NLRP3 and NLRP3-inflammasome-associated proteins (ASC, CASP1, TXNIP) are upregulated in human psoriasis skin relative to healthy skin and inhibition of NLRP3 in HaCaT cells in vitro mitigates inflammation. Further, Nlrp3 is upregulated in the skin of mice treated with imiquimod, a well-described murine psoriasis model [23]. Thus, targeting the NLRP3 inflammasome could in addition provide an explanation for the efficacy of DMF in psoriasis.

### DMF targets glycolysis

DMF and MMF succinate and inactivate glyceraldehyde 3-phosphate dehydrogenase (GAPDH) with concomitant downregulation of aerobic glycolysis in activated myeloid and lymphoid cells [24]. GAPDH inhibition may underlie or participate in the observed suppression of EAE by DMF, since heptedilic acid, a known specific GAPDH inhibitor, also attenuated the EAE in mice. Nevertheless, it was not investigated whether a cocktail of heptedilic acid and DMF could confer a synergistic effect, which would pinpoint to multiple mechanisms of action of DMF in EAE, including the described inhibition of GSDMD by DMF shown to alleviate the symptoms of EAE [15]. Depression of aerobic glycolysis by DMF has also been demonstrated in pancreatic cells [25] while molecular modeling suggested that DMF binds to the active site of methylenetetrahydrofolate dehydrogenase, cyclohydrolase and formyltetrahydrofolate synthetase 1 (MTHFD1) to inhibit pancreatic cell growth [25].

The link between DMF and glycolysis could be more complex and could depend on the cell type. In endothelial cells, DMF upregulates glycolysis and diminishes cellular respiration (oxidative phosphorylation), while it downregulates the serine and glycine biosynthesis pathway by direct inhibition of phosphoglycerate dehydrogenase (PHGDH) activity by a yet unknown mechanism. Cys369 in PHGDH can react with electrophilic molecules but it was not studied whether it is succinated by DMF [26].

### DMF inhibits cathepsin C

DMF and MMF have been found to covalently inhibit cathepsin C, most likely by modifying the active site Cys234. In the EAE model, DMF administration resulted in reduction of the activity of cathepsin C and its downstream target granzyme B in the central nervous system (CNS) on day 33 from EAE protocol initiation [27]. Thus, inactivation of cathepsin C may also contribute to the beneficial effect of DMF in EAE.

### DMF as agonist

DMF and MMF were shown to act as agonists of the hydroxycarboxylic acid receptor 2 (HCAR2), and this action resulted in reduction of neuronal deficit, inflammation and demyelination in the spinal cord of EAE mice. These biological actions of DMF were abolished in *Hcar2*^*−/−*^ mice [28]. Very recently, it was shown that in EAE, the improvement of neurological deficit by DMF was lost when the mice were subjected to lauric acid-rich diet. On the other hand, in high fiber diet, the beneficial effect of DMF in EAE was more pronounced [29]. These findings add diet as another previously unreported dimension that could affect the outcome of DMF treatment and may answer the question why DMF shows higher efficacy in some patients relative to others.

### Other targets of DMF

Various other proteins can be succinated by DMF. Recent studies have shown that the targetome of DMF comprises over 2,400 succinated Cys residues found in approximately 1,500 proteins in T cells [9] and over 4,000 succinated Cys residues in human plasmatoid dendritic cells treated with DMF [30]. Whether succination of the identified sites is linked to function(s) is mostly unknown except for a limited number of proteins/sites, which are summarized in Table 1. The DMF targetome revealed some interesting targets, as for example the enzyme adenosine deaminase (ADA) that is modified by DMF at Cys75 [9]. Mutations in residues Gly74 and Arg76 which span the Cys75 are associated with immunosuppression in humans [31]. Since Cys75 is near to these important residues (Gly74 and Arg76), it is possible that succination of Cys75 may affect the function of the ADA. This merits future investigation.

The classical indicator of NRF2 activation is the induction of *NQO1* and *HO1* genes. However, only *NQO1* was found upregulated in blood mononuclear cells obtained from DMF-treated RRMS patients in the clinical trials “DEFINE” and “CONFIRM” [32]. Currently, there is no direct evidence that NRF2 activation is responsible for the therapeutic efficacy of DMF in RRMS. The participation of the above-mentioned alternative mechanisms/pathways of DMF action (identified in EAE mouse models or with in vitro experiments) in human RRMS patients treated with DMF should be tested in clinical specimens. For example, this could be carried out by determining the succination status of the DMF targetome in clinical specimens, in combination with measurement of the changes in the expression of downstream targets or the presence of novel protein fragments such as the GSDMD-N. In turn these will aid in the delineation of the molecular mechanisms that account for the therapeutic effect of DMF in humans.

## Endogenous succination

Endogenous succination is highly enhanced by loss-of-function mutations in the gene encoding for fumarate hydratase (FH). FH is an enzyme involved in the Krebs cycle and its absence causes intracellular accumulation of fumarate at mM levels. This predisposes for an aggressive form of kidney cancer known as hereditary leiomyomatosis and for renal cell carcinoma (HLRCC). FH catalyzes the reversible addition of water to fumarate to yield malate. The role of endogenous succination has been reviewed recently [33]. Endogenous succination of proteins takes place because of the huge intracellular fumarate concentrations in HLRCC. Depletion of FH has also been found in tumors, and increased fumarate in the interstitial fluid of tumors can induce succination of ZAP70 at Cys96 and Cys102, thus, suppressing the activation of CD8^+^ T cells [34].

Reduction of NADH re-oxidation results in inhibition of Krebs cycle and this, in turn, induces protein succination. In accordance, in the NADH dehydrogenase Fe-S protein knockout (*Ndufs*^*−/−*^) mouse model of Leigh syndrome, protein succination is increased in the brainstem of mice [35]. This could point to increased succination in the brainstem of human patients with Leigh syndrome. Identification of proteins targeted by endogenous fumarate is important not only to understand the pathology of HLRCC but also to identify proteins that are susceptible to succination. These proteins may then be tested as potential targets of DMF.

## Potential applications of DMF in disease treatment

The biochemical effects of DMF discussed, so far, have attracted significant interest in repurposing the compound for the treatment of diverse pathological conditions. In the following sections, we review the latest findings on the therapeutic efficiency of DMF and its potential clinical applications for highly prevalent diseases.

### Neurodegenerative diseases (other than MS)

Recent preclinical studies indicate that DMF may attenuate neurodegeneration, in several animal models of neurodegenerative diseases. In streptozotocin (STZ)–induced sporadic Alzheimer’s disease (AD) rats, DMF protected the hippocampus and basal forebrain and improved spatial memory, while its effects on neuropathological features were modest [36]. Also, reduced lymphocyte counts and lower serum IL-6 and IFN-γ, together with enhanced neurogenesis and brain-derived neurotrophic factor (BDNF)-dependent neuroprotection were shown [37, 38]. Consistent with these findings, astrocytic NRF2 signaling was activated in the *App*^NL−G−F^ (APP-KI) AD mouse model, reducing neuroinflammation and cognitive decline by modulating astrocyte-microglia crosstalk through the complement C3–STAT3 pathway [39]. DMF may also provide neuroprotection, as shown in preclinical models of Parkinson’s disease (PD). In 6-hydroxydopamine (6-OHDA)-lesioned rats, DMF reduced dopaminergic neuron loss, preserved striatal dopamine levels, and attenuated astrogliosis and microgliosis, a function that was associated with NRF2 activation [40]. These effects are likely linked to the DMF's ability to maintain glutathione and dopamine transporter levels, while lowering 3-nitrotyrosine, α-synuclein oligomers, NF-κB, and other oxidative and inflammatory markers [41]. In rotenone-treated mice, a PD murine model, DMF activated the NRF2 pathway that reduced cathepsin D and restored autophagic flux and inhibited apoptosis [42]. Mitophagy, a selective mitochondrial autophagy, is crucial for mitochondrial and neuronal homeostasis. Accordingly, it was found that DMF enhanced mitophagy via the NRF2/BNIP3/PINK1/Parkin pathway, leading to behavioral improvements in a PD mouse model [43]. Finally, DMF exerts beneficial effects in the Tg-*SOD1*^*G93A*^ mouse model of human *SOD1*-Amyotrophic Lateral Sclerosis (ALS) [44]. In the above-mentioned cases, it appears that the therapeutic potential of DMF is directly linked to its ability to activate the NRF2 pathway. However, the presence of additional mechanisms cannot be excluded.

### Cardiovascular diseases (CVDs)

Over recent years, several studies have reported that DMF exhibits multiple cytoprotective activities that counteract key processes underlying CVDs. In experimental hypertension models, DMF increased *Nrf2* mRNA expression, lowered plasma asymmetric dimethylarginine (ADMA), and attenuated renin-angiotensin signaling. These changes are associated with enhanced nitric oxide bioavailability and upregulation of autophagy and mitochondrial regulators, such as Ulk1, PGC-1α, and Atg5 [45]. Additionally, DMF’s antihypertensive effects have been linked to modulation of genes involved in vascular tone regulation, including TET1 and KCNMB1. Nevertheless, in this study, the efficacy of DMF was similar to succinic acid and no synergistic action between DMF and succinic acid was found. Since succinic acid is not a Michael acceptor, this study likely suggests that DMF does not act through its classical electrophilic reactive character [46]. In stroke and ischemia animal models, DMF reduced infarct size by boosting NRF2 and HO-1 expression, lowering the proinflammatory cytokines IL-1 and TNF, suppressing NF-κB, and reducing T-cell and neutrophil infiltration [47–49]. In high cholesterol diet-induced atherosclerosis models, DMF activated NRF2, decreased aortic oxidation, and lowered total serum cholesterol, triglycerides, and LDL cholesterol [50]. Clinical evidence supports these findings, showing that DMF therapy in moderate-to-severe plaque psoriasis increases the serum atheroprotective cytokine adiponectin and reduces apolipoprotein B and total cholesterol [51]. Similarly, in multiple sclerosis patients, DMF treatment elevates HDL cholesterol and improves HDL-to-LDL and HDL-to-total cholesterol ratios without increasing LDL levels [52].

### Eye diseases

DMF is a promising candidate for multiple eye diseases, supported by evidence from various experimental and clinical studies. It has shown efficacy in mitigating optic neuritis, a common demyelinating symptom in MS patients, through systemic administration that preserves retinal nerve fiber and ganglion cell layers. This effect occurred independently on induction of NRF2 target genes [53], in accordance with all the above-mentioned data, supporting the NRF2-independent action of DMF in RRMS. Further, in vivo studies have demonstrated the neuroprotective effects of DMF in optic nerve injury and light-induced retinal degeneration by preserving retinal ganglion cells and photoreceptors [54, 55]. Two pilot human studies reported improvements in uveitis and cystoid macular edema symptoms following DMF treatment [56, 57]. In diabetic retinopathy models, DMF decreases retinal inflammatory markers, such as inducible nitric oxide synthase (iNOS) and cyclooxygenase-2 (COX-2), while inducing HO-1, suggesting attenuation of inflammation and oxidative stress [58]. A phase II clinical trial is underway to evaluate the safety and efficacy of DMF in geographic atrophy associated with age-related macular degeneration (AMD), a condition characterized by progressive loss of photoreceptors and retinal pigment epithelium (RPE), with oral doses administered over the course of one year (NCT04292080).

### Pulmonary diseases

Preclinical evidence from several models of pulmonary arterial hypertension and lung fibrosis suggests that DMF is protective. In chronically hypoxic mice, DMF mitigated lung oxidative stress and inflammation and exerted protective effects in pulmonary arterial smooth muscle cells and human fibroblasts. This action was mediated through suppression of multiple pathways, *i.e.,* by blocking the inflammatory response and by blocking the pro-fibrotic response independent of NRF2 activation [59]. DMF was also effective in an age-dependent persistent lung fibrosis model [60]. In bleomycin-induced non-resolving fibrosis, daily inhaled DMF for 3–6 weeks restored NRF2, reduced oxidative stress, and promoted fibrosis resolution, whereas oral administration was ineffective [60]. In idiopathic pulmonary fibrosis, inhaled ROS-responsive DMF liposomes reduced fibrosis via NRF2 activation [61]. Similarly, DMF-encapsulated nanoparticles attenuated pulmonary dysfunction and clinical signs of EAE [62]. Emerging data indicate potential benefit in airway diseases. Anecdotal reports describe symptom improvement in psoriasis patients with asthma [63]. In allergic asthma, intranasal DMF given before house dust exposure abrogated airway inflammation, mucous metaplasia, and hyperresponsiveness by impairing dendritic cell migration and attenuating Th2 responses [64]. In acute lung injury induced by intratracheal lipopolysaccharide, a single intraperitoneal DMF dose reduced pulmonary edema and inflammation [65]. In diesel exhaust particle exposure, DMF lowered lung injury, inflammation, and oxidative stress [66]. In TNF-α-induced systemic inflammatory response, DMF suppressed lung, cecum, and uterus lesions and reduced cytokines by inhibiting TNF-α-triggered necroptosis, suggesting a novel mechanism [67]. In viral infections, DMF induced an interferon-independent antiviral program effective against HSV-1/2, Zika, and SARS-CoV-2, most likely through reactivation of the NRF2 that is downregulated in the above-mentioned viral diseases [68]. Its potential use in COVID-19 is supported by meta-analyses and real-world studies in MS patients showing reduced risk for severe disease [69, 70]. Descriptive studies confirmed that most MS patients on DMF experienced mild, non-hospitalized COVID-19 symptoms [71, 72]. Taken together, these findings suggest that DMF exhibits a strong immunomodulatory effect in pulmonary diseases.

### Cancers

The antitumoral potential of DMF has been investigated in several cancer types. Its antiproliferative effects are primarily linked to regulation of NF-κB nuclear translocation, activation of NRF2, ERK1/2 and p38 MAPKs, while suppression of metastasis involves inhibition of matrix metalloproteinases (MMPs) and of very late antigens (VLAs) [73, 74]. Initial evidence came from melanoma models, where daily DMF significantly reduced tumor growth, mean tumor volume and metastasis [75]. Successive studies confirmed these results with DMF administered alone [76] or in combination with dacarbazine or vemurafenib, leading to further inhibition of tumor growth, delayed metastasis, impaired cell migration, and improved overall survival [76]. In colon cancer, early studies demonstrated efficacy in azoxymethane-induced aberrant crypt foci, with DMF reducing foci yield and invasive adenocarcinoma incidence [77, 78]. Later work showed DMF cytotoxicity in several cancer lines and confirmed in vivo efficacy in two colon cancer models, where DMF reduced tumor occurrence and growth by dampening chronic inflammation [73]. Moreover, DMF enhanced the antitumor activity of mitomycin C in colon cancer cells [79] and in breast cancer cells [80]. In addition, DMF impaired breast tumor growth in vitro and in vivo, through covalent modification of p65 at Cys38, which prevents the nuclear localization of p65 [81]. Clinical evidence for the efficacy of DMF in cancer, includes a phase II randomized trial in glioblastoma multiforme, where DMF administered before surgery and standard therapy improved Karnofsky performance status [82]. A phase I trial confirmed the safety of DMF in combination with radio- and chemo-therapy [83].

Other tumors for which DMF may have beneficial effects include cutaneous T-cell lymphoma (CTCL), non-small cell lung cancer (NSCLC), hepatic cancer, acute myeloid leukemia (AML), and oral squamous cell carcinoma (OSCC). In CTCL models, DMF delayed tumor growth, prevented metastasis and induced tumor cell death. These findings were later confirmed in a phase II clinical study (NCT02546440) [84, 85]. In NSCLC, DMF suppressed tumor growth and proliferation [86]. In a two-stage chemical hepatocarcinogenesis model, DMF improved body weight, liver histopathology, DNA damage, and antioxidant/inflammatory pathways likely through NRF2 activation and NLRP3 inflammasome inhibition [87]. In acute myeloid leukemia (AML) xenografts, DMF combined with vitamin D derivatives markedly enhanced tumor suppression compared to single treatments [88]. In oral squamous cell carcinoma (OSCC), DMF reduced tumor mass and neutrophilic infiltration, by increasing caspase 3, HO-1 and MnSOD expression [89]. Taken together, these findings suggest that DMF exerts an anticancer activity.

## Other skin diseases

In a case study, DMF was found beneficial in the treatment of Papillon-Lefevre syndrome (PLS) that is due to inactivating mutations in the gene encoding for cathepsin C. This was accidentally noticed when a female patient suffering from PLS showed signs of MS and was treated with DMF [90].

Recently, DMF has been tested in a small number of patients for the treatment of other granulomatous and inflammatory skin diseases, specifically, for granuloma anulare, cutaneous sarcoidosis, lichen planus, pityriasis rubra pilaris, and chronic discoid lupus erythematosus (CDLE). The results were promising, since most patients exhibited complete clearance and the rest exhibited partial response. One patient suffering from CDLE showed stable disease on DMF in combination with hydroxychloroquinone [91]. This finding, in combination with the limited side-effects that could be eliminated through DMF dose adjustments, are promising and may encourage validation of DMF for the treatment of other skin diseases in future clinical trials.

Interestingly, tofacitinib, a JAK inhibitor, was found effective in managing the severe epidermal disorder, Netherton syndrome (NS), as indicated in a case report [92]. Other case reports with upadacitinib and abrocitinib administration in NS patients showed improvements or slight initial improved or in one case report no improvement [93]. Also, stable expression of the constitutively active NRF2 in the *Spink5*^*−/−*^ mouse model of NS promoted the stabilization of corneodesmosomes and prevented premature desquamation, while it partially alleviated the constitutive epidermal inflammation [94]. Given the fact that DMF activates NRF2 and inhibits JAKs, it could exert this dual action in NS and it worths to be repurposed for treatment of NS.

## DMF for the treatment of other diseases

GSDMD is linked to familial Mediterranean fever (FMF) [15]. FMF is the most common monogenic autoinflammatory disease characterized by recurrent episodes of fever and abdominal pain. FMF is due to inactivating mutations in the *MEFV* gene that activates the pyrin inflammasome. The *Mefv*^*V726A/V726A*^ mice recapitulate human FMF and deletion of the *Gsdm* gene rescues the *Mefv*^*V726A/V726A*^ phenotype [95]. Therefore, targeting GSDMD could provide a new therapy for FMF. Given the fact that DMF succinates and inactivates GSDMD, it is logical to assume that DMF could be a new pharmaceutical compound of choice for treatment of FMF. Indeed, administration of DMF in *Mefv*^*V726/V726A*^ mice alleviated the disease symptoms [15]. A survey in the clinical trials database with “dimethyl fumarate” as query (https://clinicaltrials.gov, September 2025) identified a total of 140 clinical trials with most of them involving either MS or psoriasis. Nevertheless, there are certain clinical trials that enroll patients beyond the classical applications of DMF. Table 2 shows other diseases treated with DMF in clinical trials, thus highlighting the multidimensional therapeutic potential of DMF.

**Table 2:** Clinical trials highlighting the multidimensional therapeutic character of DMF

Disease	Number of trials	Trial characterization	Clinical trial number
Glioblastoma multiforme	1	Completed	NCT02337426
Cutaneous lupus erythematosus	1	Completed	NCT01352988
Active rheumatoid arthritis	1	Completed	NCT00810836
Obstructive sleep apnea	1	Completed	NCT02438137
Cutaneous T cell lymphoma	1	Completed	NCT02546440
Alzheimer’s disease	1	Recruiting	NCT06850597
Adrenomyeloneuropathy	1	Recruiting	NCT06513533
Geographic area associated-age-related macular degeneration	1	Recruiting	NCT04292080
Acute ischemic stroke	1	Terminated	NCT04890353
Relapsed Refractory CLL/SLL	1	Terminated	NCT02784834
Systemic sclerosis-associated pulmonary arterial hypertension	1	Terminated	NCT02981082

## Potential side effects and concerns with DMF

To this end, the potential side effects of DMF should also be considered. In RRMS patients, DMF administration has been linked with increased risk for progressive multifocal leukoencephalopathy (PML). PML is rare opportunistic infection by the John-Cunnigham virus (JCV) that is potentially fatal. DMF was shown to induce mild lymphopenia in a subset of patients [96], which in turn increases the probability for PML. The PML incidence between DMF administered patients is 0.02 per 1000 patients [97]. Thus, it is necessary for patients under DMF treatment to be regularly monitored for lymphocyte counts. Adjustment of the dose may also be required to alleviate the side effects.

DMF has also been reported to be a potent irritant. Indeed, DMF sachets are added in furniture as fungicides and have been found to cause allergic contact dermatitis [98]. Also, delayed hypersensitivity to DMF has been reported in RRMS patients treated with DMF [99]. These situations highlight the need to include DMF testing in the patch tests used for allergen identification. Finally, future studies should aim to delineate the mechanisms of DMF-induced contact dermatitis and delayed hypersensitivity.

## Conclusions

Initially, it was believed that DMF is a prodrug with MMF being its active metabolite. Based on chemical reactivity, the ability to cross cellular membranes, and newer high-throughput mass spectrometry data, DMF has been identified as the pharmacologically active compound. The successful application of DMF in the treatment of RRMS and psoriasis points to its multidimensional therapeutic potential. It is, therefore, tempting to investigate whether DMF could be additionally applied to treat other diseases. In this direction, new preclinical studies in animal models and recent clinical trials revealed potential therapeutic effects of DMF in diseases like pulmonary and eye diseases and cancer.

**Fig. 4:**
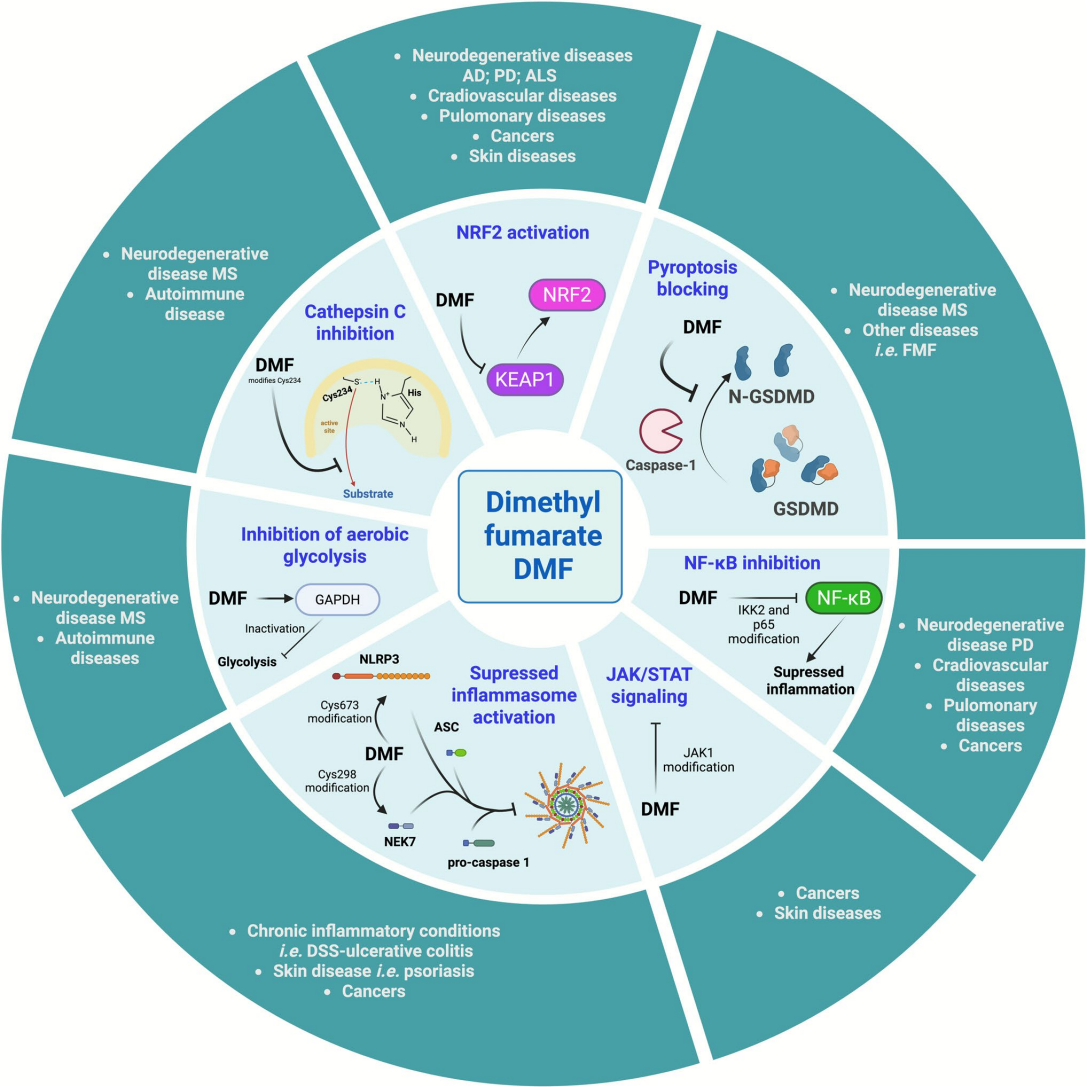
Synopsis of the DMF dependent mechanisms of action and their disease connections. Created in BioRender. Zingkou, E. (2026) https://BioRender.com/l3c3kjd

In accordance, the described DMF targetome was found to be more diverse and complicated than previously thought as summarized in Fig. 4. Deciphering the complete DMF targetome will result in reevaluation of the DMF efficacy in treatment of other diseases than those it was originally approved for. For example, DMF was found to target GSDMD, but GSDMD is a key target for the treatment of FMF. Thus, DMF may be repurposed for FMF [15]. Targetome identification will also aid the development of more specific drugs for treatment of severe disease will potentially minimally side effects. To this end, new computational approach could aid in the identification of potential succination sites that can be verified experimentally [100].

## Data Availability

All data are included in the manuscript.
